# Investigating the fluid and electrolyte prescribing knowledge of Foundation Year doctors

**DOI:** 10.1308/rcsann.2025.0004

**Published:** 2025-03-25

**Authors:** D Johnson, J Houdmont, N Levy, DN Lobo

**Affiliations:** ^1^ West Suffolk NHS Foundation Trust, UK; ^2^ University of Nottingham, UK; ^3^ Nottingham University Hospitals NHS Trust, UK; ^4^ University of Pennsylvania, Philadelphia, USA

**Keywords:** Education, Fluid and electrolytes, Intravenous, Knowledge, Prescribing, Trainee doctors

## Abstract

**Introduction:**

Changes to the medical curriculum have been advocated to improve knowledge on fluid and electrolytes. We aimed to determine the contemporary level of knowledge of trainee doctors on different aspects of fluid and electrolyte prescribing.

**Methods:**

An online survey was distributed to Foundation Year doctors working throughout the United Kingdom. The first part determined demographic information, where participants studied and currently work, and probed their perceptions of their knowledge on fluid and electrolytes. The second part tested knowledge on a wide variety of aspects of fluid and electrolyte management using 20 multiple-choice questions.

**Results:**

In total, 190 responses were received. Trainee doctors remain responsible for much of the fluid and electrolyte management of patients, and often practise independently. Overall, the average ‘score’ of each respondent was suboptimal (52%), with no significant difference found between doctors in the first or second year of postgraduate medical practice. Many participants were unable to correctly identify the components of common intravenous fluid products. Understanding of daily electrolyte requirements was also demonstrated poorly, although most showed a good understanding of the daily requirements of water and glucose. The amount of time in medical school allocated to the topic remains low, as do doctors' confidences in their abilities to prescribe fluid and electrolytes.

**Conclusions:**

Knowledge surrounding fluid and electrolyte prescribing remains suboptimal, and Foundation Year doctors are frequently undertaking this responsibility independently. These findings reflect previous research performed over the past 20 years, and little improvement appears to have been made.

## Introduction

The prescription of fluids and electrolytes constitutes a significant part of the management of hospital inpatients and is a task frequently delegated to the most junior members of the medical team. Foundation Year doctors (within the first two years of undergraduate qualification) do the majority of all inpatient prescribing, including that of intravenous fluid and electrolytes.^1–5^ However, although most prescriptions for medications are routinely screened by ward pharmacists, prescriptions for intravenous fluids often are not.^6^ This, accompanied by the fact that fluid balance charts are often not checked during ward rounds, means that the most junior medical members of the team often manage patients' fluid and electrolyte statuses independently.^3,4^

It has previously been reported that doctors in training may lack the requisite knowledge about fluid and electrolyte management to be shouldering this responsibility.^2–5,7^ This paucity of knowledge is not without risks. The UK National Confidential Enquiry into Patient Outcome and Death in 1999 reported that “fluid imbalance can contribute to serious postoperative morbidity and mortality”.^2^ Patients who are prescribed excessive intravenous fluids may develop salt and water overload, leaving them at increased risk of its associated complications, which include tissue oedema, anastomotic breakdown and pulmonary oedema, whereas those patients who are not prescribed enough may suffer the consequences of hypoperfusion and end organ dysfunction, including acute kidney injury.^8–11^ Likewise, electrolyte mismanagement may lead to electrolyte disturbances, which may in turn contribute to clinical features such as confusion, prolonged postoperative ileus and cardiac arrhythmias.^9,10,12–15^ Other lesser-known risks are also becoming increasingly apparent, such as the risk of hyperchloraemic metabolic acidosis secondary to excessive infusion of 0.9% sodium chloride solution.^9,12,16^

This survey aimed to assess the contemporary knowledge of Foundation Year doctors with regard to the safe and effective prescribing of fluids and electrolytes.

## Methods

Part one of this survey established participants' demographics, educational and professional backgrounds (e.g. location of study and current employment), and perceptions about their education and knowledge concerning fluid and electrolyte prescribing. Part two assessed participants' knowledge with regard to safe and effective fluid and electrolyte prescribing. Multiple-choice questions with one correct answer and five distractors (Appendix 1 – available online) were developed largely to probe knowledge of the guidance from the National Institute for Health and Care Excellence (NICE) on fluid and electrolyte prescribing.^17^ Questions were also drawn from other sources, with permission.^3,10^

The questionnaire was distributed largely via the medical education departments of numerous hospitals in the United Kingdom. Social media was also used to a lesser extent to offer participation. Responses were collected from 1 August 2023 to 15 May 2024.

The protocol for the study was approved by the University of Nottingham Faculty of Medicine & Health Sciences Research Ethics Committee (Ethics reference number FMHS 252-0423). All participants provided informed consent having read the participant information sheet on the first page of the questionnaire by ticking a box at the bottom of the page, which was required to move on to the main questionnaire.

The questionnaires were completed online anonymously using the University of Nottingham's Online Surveys licence (https://www.onlinesurveys.ac.uk), which is a secure online survey platform (https://www.onlinesurveys.ac.uk/help-support/online-surveys-security/). The data were collated by the online survey platform and populated into a database that was used for analysis. Access to the collated database was restricted to those personnel approved by the chief investigator and recorded as such in the study records.

Data were analysed using SPSS statistics software v25 (IBM Corp., Armonk, NY, USA) and Microsoft Excel 365 (Microsoft® Corp., Redmond, WA, USA).

## Results

Overall, 190 responses were received, but the method of dissemination of the survey invitation meant we were unable to determine the response rate. Of these responses, 115 (61%) were from doctors in their first year of foundation training (FY1) and 75 (39%) responses were from those in their second year (FY2). The majority (158, 83%) underwent a standard undergraduate medicine course, although 32 (17%) received their medical education via a truncated graduate-entry medicine course. A breakdown of where participants were working at the time of the survey and where they attended medical school can be found in Tables 1 and 2.

**Table 1:** In which region do you currently work?

Location	Respondents
London	42
Midlands and East	46
North	52
South East	24
South West	13
Northern Ireland	2
Scotland	1
Wales	10

**Table 2:** Where did you attend medical school?

Location	Respondents
London	42
Midlands and East	47
North	40
South East	16
South West	6
Northern Ireland	5
Scotland	7
Wales	7
Italy	2
Cyprus	3
Ireland	4
Poland	2
Unspecified	3

The countries of Pakistan, Kazakhstan, India, China, Bahrain and Lebanon, each received 1 response (total 6)

Most respondents (110, 58%) reported working in a district general hospital at the time of survey engagement. A further 72 (38%) reported working in a main teaching/tertiary hospital, and 7 (4%) reported working in the community (e.g. in a hospice, psychiatric unit or general practice surgery). None were working in private practice, and one respondent did not answer.

When asked to recall the number of hours of teaching on fluid and electrolytes participants had received at medical school, 72 (38%) reported more than four hours. By contrast, nearly half (85, 45%) reported less than four hours of teaching on the subject. The remainder (33, 17%) responded ‘no idea’.

The majority (139, 73%) of participants claimed to be ‘somewhat confident’ in their ability to safely prescribe fluid and electrolytes, 37 (19%) reported feeling ‘very confident’ and 13 (7%) reported feeling ‘not confident’. Only 74 (39%) reported receiving guidance in any form regarding fluid and electrolyte prescribing since starting on their current rotation/job.

Respondents reported that doctors in training (defined herein as any doctor who is not a consultant) were responsible for the vast majority of fluid and electrolyte prescriptions, whether for patients with uncomplicated or complicated (subjectively defined) fluid requirements (Tables 3 and 4).

**Table 3:** Who is normally responsible for prescribing fluid and electrolytes on your ward for patients with uncomplicated fluid requirements?

Grades	Respondents, *n* (%)
FY1	70 (36.8)
FY2	4 (2.1)
CT	1 (0.5)
ST	0
Consultant	0
Juniors shared (FY1, FY2, CT and ST)	97 (51.1)
All doctors shared	18 (9.5)
Other	0

CT = core trainee; FY = Foundation Year; ST = specialty trainee

**Table 4:** Who is normally responsible for prescribing fluid and electrolytes on your ward for patients with complicated fluid requirements?

Grades	Respondents. *n* (%)
FY1	27 (14.2)
FY2	8 (4.2)
Core trainee	11 (5.8)
Specialty trainee	12 (6.3)
Consultant	4 (2.1)
Juniors shared	81 (42.6)
All doctor shared	46 (24.2)
Other	1 (0.5)

FY = Foundation Year

When asked if a fully registered healthcare professional (FY2 and above, pharmacist, etc.) routinely checked the fluid and electrolyte prescriptions of FY1s on the ward, the majority (103, 54%) responded ‘no’, 17 (9%) responded ‘yes’ and the remainder were ‘unsure’. The majority (103, 54%) reported that patients' fluid balance charts were not checked during the daily morning ward round, whereas 81 (43%) reported that they were. A small number (6, 3%) reported that they were checked daily, but after the ward round.

**Figure 1:**
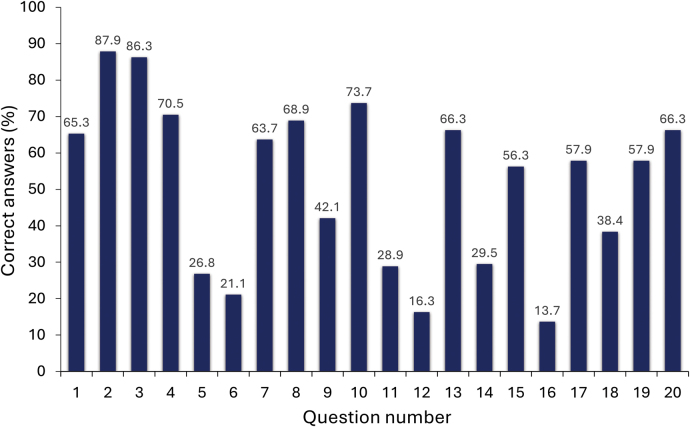
Percentage of correct responses to the 20 knowledge-based questions

The percentage of correct answers to the 20 knowledge-based questions is shown in Figure 1. A test ‘score’ for each participant was also calculated (percentage of answers correct for these 20 questions). Overall, participants received a mean score of 52% (median 50%). There was no significant difference in mean test score between FY1s and FY2s (52% vs 51%, *p *= 0.46). Total scores were also similar by region of work, with no significant difference in average score among the top six regions. The region of medical training also appeared to have no bearing on average test score with no significant difference found among the top four regions by number of respondents.

Responses to the questions in part two of the questionnaire were varied. The vast majority (86%) of respondents correctly identified the standard initial volume of crystalloid recommended for intravenous fluid resuscitation (in a patient >50kg without any past medical history) as 500ml. A similarly large proportion (88%) was also able to correctly identify the daily water and glucose maintenance requirements for a patient who was ‘nil-by-mouth’: 25–30ml of water; 50–100g of glucose. A smaller majority (65%) correctly identified the daily electrolyte requirements for such a patient (1mmol/kg of sodium, potassium and chloride).

Awareness of the molar concentrations of electrolytes in commonly used intravenous fluids was low. Although 70% of respondents correctly identified the component breakdown of 0.9% sodium chloride, only 42% could do the same for Hartmann's solution. Around three-quarters (74%) correctly reported the weight of glucose in 1L of 5% dextrose.

Few respondents (38%) were aware of the risk of metabolic acidosis associated with over-infusion of 0.9% sodium chloride solution, and even fewer (30%) were able to correctly identify the proportion of a person's guided daily allowance of salt that can be found in 1L of this solution.

Knowledge of the significance of certain electrolyte imbalances was varied. Although some 66% and 56% of respondents correctly identified that hypophosphataemia and hypokalaemia are components of refeeding syndrome, only 30% were aware of the association between hypomagnesaemia and atrial fibrillation. Around half (58%) of respondents correctly identified the typical electrolyte imbalance (hypokalaemic, hypochloraemic metabolic alkalosis) associated with excessive vomiting. Around two-thirds (66%) were able to identify the correct equation for calculating the anion gap.

Knowledge on fluid and electrolyte monitoring was similarly varied. Most respondents (71%) were aware that the minimum hourly urine output desired is >0.5ml/kg/h (as a urine output below this denotes a common criterion for acute kidney injury), and that urea and electrolytes should be measured daily when initiating intravenous fluids (64%). However, only 27% correctly identified ‘daily weights’ as the most accurate serial measure of fluid balance, and even fewer (21%) were aware of NICE guidance that recommends serum chloride concentrations to be initially measured every 24h when managing a patient with 0.9% sodium chloride solution.^17^ Knowledge of the passive leg-raising test as a tool for assessing fluid status was also poor with only 16% of respondents showing a clear understanding of how it is performed.

Awareness that living alone/social deprivation is a significant risk factor for salt and water depletion in the older adult was demonstrated by 58% of respondents.

The question on the volume of intravenous maintenance fluid to be given over a 24-h period for a hypothetical obese male patient who is nil-by-mouth aimed to test awareness of NICE guidance that states ideal body weight should be used when determining a patient's daily fluid requirements.^17^ The weight, height and body mass index (BMI) for the patient were provided in the question. This question was answered poorly, with only 14% answering correctly (2,000ml for a patient weighing 115kg, height 183cm, BMI 34kg/m^2^). The most common response appeared to reflect the use of actual body weight for calculation of maintenance intravenous fluid volume. A quarter of respondents stated they would have prescribed 3,400ml.

## Discussion

This survey reaffirms previous findings that Foundation Year doctors undertake a significant proportion of the prescribing of fluid and electrolytes for inpatients. This work is frequently performed without review of the prescriptions by senior doctors or pharmacists.

Despite this high level of responsibility only 19% of respondents reported feeling ‘very confident’ in their ability to ‘prescribe fluid and electrolytes safely’. This perceived unpreparedness may stem from medical school, with only 38% of participants reporting more than four hours of teaching for this fundamental and clinically ubiquitous topic throughout the entirety of medical school. The situation is further compounded by the fact that only 39% of respondents reported receiving guidance on fluid and electrolyte management since starting their current job. These shortcomings do not go unnoticed considering a majority of consultants believe their trainee doctors are inadequately trained in fluid and electrolyte prescribing.^4^

The proportion of respondents correctly indicating the minimum desired hourly urine output as 0.5ml/kg/h was 71%. Only a small minority demonstrated good knowledge of what constitutes a passive leg-raising test (16%), and when asked for the most accurate measurement for serially assessing fluid balance, only 27% offered daily-weighing of the patient. Instead, the most popular response was ‘urine output over 24 hours’, which mirrors a previous survey that asked this question.^3^ Given that nearly one-third of doctors were unable to correctly identify the desired minimum hourly urine output, the use of ‘urine output over 24 hours’ by a majority of doctors to determine a patient's fluid balance, and hence further prescription of fluids, may constitute a recipe for fluid mismanagement.

The question in our survey with the lowest rate of correct response (14%) was question 16 which asked the participants for the standard 24-h volume of maintenance fluid they would prescribe for an obese patient who is nil-by-mouth. This highlights that the principle of using ideal body weight for obese patients when determining the volume of required maintenance water is sparsely known among Foundation Year doctors, and that members of this patient group may be at a uniquely high risk of iatrogenic fluid overload and its potentially harmful consequences.

For electrolyte management, a small majority (64%) indicated that they would measure urea and electrolytes daily in those being initiated on intravenous fluids and electrolytes. However, very few were aware of the risk of hyperchloraemic metabolic acidosis as a consequence of excessive 0.9% sodium chloride infusion, and the subsequent NICE recommendation that those receiving it should initially have their serum chloride concentrations measured daily.^17^ The fact that this risk is known only to a small minority suggests it may not be a risk that is incorporated into the limited teaching time allocated to undergraduate training on fluids and electrolytes.

More than half (65%) of respondents were able to correctly identify the daily maintenance electrolyte requirements of potassium, sodium and chloride (1mmol/kg of each). This may show improvement compared with a survey from 2001, but is generally in alignment with more recent investigations, suggesting that there may have been some educational improvement on this fundamental point over time.^3,^^18^

Just over half of our respondents were aware that hypokalaemia and hypophosphataemia are components of refeeding syndrome. In a similar vein, the increased risk of atrial fibrillation associated with hypomagnesaemia was poorly recognised (30% of respondents). Once again, these shortcomings in knowledge are made potentially more significant by the independence with which Foundation Year doctors appear to be managing the electrolyte status of inpatients.

Perhaps, the most fundamental information one might expect a doctor to possess is the contents of the bags of fluids they prescribe. Our survey suggests a proportion of Foundation Year doctors were unaware of the molar concentrations sodium and chloride (154mmol/L of both) in 0.9% sodium chloride. Furthermore, 7.4% of respondents suggested that this solution contains 5mmol/L of potassium. A similarly significant minority was unable to correctly report the mass of glucose in a 1-L bag of 5% dextrose (glucose) solution (despite being given its concentration of 5%). The majority of participants were unable to correctly identify the concentrations of sodium, chloride and potassium in Hartmann's solution. Although still being suboptimal, these numbers do appear to reflect an improvement compared with previous studies of doctors. For example, a previous study revealed only 31% of its FY1 respondents knew the sodium content of 0.9% sodium chloride solution.^18^

Interestingly, these numbers also appear higher than those found in a survey of final/penultimate-year medical students, only 59% and 20% (vs 69% and 42% in this survey of doctors) of whom correctly identified the components and concentrations of 0.9% sodium chloride and Hartmann's solution, respectively.^19^ This may suggest that time elapsed since undergraduate medical training does not explain the below par knowledge found in the literature, and that this may be knowledge gained to some degree after starting work as a doctor.

### Study limitations

Although this study investigates some specific areas of fluid and electrolyte prescribing knowledge that have previously been researched, and, thereby, offers an updated and contemporary assessment, it is worth acknowledging that any apparent changes in knowledge shown over time by the few available studies could reflect differences among the studies' methods, such as alternative questioning methodologies (e.g. open-ended vs multiple-choice questioning) or differing degrees of participation from different parts of the country. Inferences made about longitudinal changes should thus be made sparingly.

Further limitations include the modest sample size compared with the overall number of Foundation Year doctors (of which there were 16,270 in 2022; e.g. General Medical Council).^20^ In addition, although our data reflect a good geographical spread of participation by doctors throughout England, only 13 respondents reported working in Wales, Northern Ireland and Scotland, combined. The applicability of our results to doctors working in these countries may therefore be limited.

Given the lack of fundamental knowledge concerning fluid and electrolyte prescribing demonstrated with this survey, an increase in formal teaching time on the topic may have a role to play in improving patient safety. This could happen both during medical school, where the number of hours dedicated to fluid and electrolytes appears to be low, and after finishing medical school as well. Specifically, the mandatory education time during the Foundation Programme could be used for such purposes.

However, as noted by the Institute for Safe Medication Practices, although “education has its place as a basic prerequisite”, on its own it is a “weak improvement strategy”.^21^ Although it may be necessary in situations in which fundamental knowledge is missing (as we have demonstrated with our survey) it should be augmented by other mechanisms to see a translation into improvements in prescribing practice and patient safety.^22^

Our survey suggests that senior supervision of fluid and electrolyte management is rare. More frequent senior input may generate opportunities to change the prescribing behaviours of Foundation Year doctors, and thereby improve practice and safeguard patients.

The implementation of clinical decision support tools has also been associated with improved prescribing safety in various settings.^23^ Such tools have been used in the realm of fluid and electrolyte prescribing in a successful attempt to increase the prescribing prevalence of balanced crystalloids over 0.9% sodium chloride.^24^ Further development and implementation of such tools to help guide fluid and electrolyte prescribing in a variety of scenarios constitutes a promising realm of future research.

## Conclusions

Foundation Year doctors appear to receive little education on fluid and electrolyte prescribing while at medical school. This may contribute to the lack of confidence reported by many in their abilities to undertake this task, which is especially important given the independent nature of their practice. Participants' perceptions of their abilities seem to be realised by the lack of knowledge demonstrated in the survey across a variety of aspects of fluid and electrolyte prescribing. While the results of this survey may suggest improvement over time in isolated areas (e.g. knowledge of the constitutions of different intravenous solutions), the level of knowledge in fluid and electrolyte prescribing shown leaves considerable room for improvement.

## Data Availability

Data are available upon reasonable request from JH (jonathan.houdmont@nottingham.ac.uk).
